# Florid Mendacity

**Published:** 1897-05

**Authors:** 


### Abstracts and Selections.

**A Lotion for Pityriasis Versicolor.**—*Lyon Medical* for November 28th, takes the following formula from the *Concours Medical* from November 4th:

℞ Corrosive sublimate..................1 part.
Oil of lavender........................4 parts.
Tincture of lavender................120 parts.
Green soap..............................80 parts.

M. Apply the liquid to the affected part and let it dry; three days later, take a bath. One application is said to be enough.

### "Florid Mendacity;" Why Physicians Do Not Advertise.

Dr. J. M. Madden, in the *Journal of the American Medical Association*, Feb. 27, 1897, scores the lay press for the encouragement they give to all kinds of frauds in the name of medicine, even to tansy wafers and other nostrums sold for criminal purposes. He sets forth a delectable dish of nastiness which is sickening to read. But take any newspaper—especially the religious papers, and you will see the picture is not overdrawn. It seems that the article is a reply to the inquiry by the proprietor of some big paper why doctors do not advertise. Dr. Madden says, *inter alia:*

Such, then, is the character of the company to which the honorable medical man is introduced, no matter how modest his announcement, if he allows his name to appear in a newspaper. Surely, it is a company from which he must shrink with loathing and disgust. Beside, what chance would the physician's modest card have to attract the attention of the public in the midst of all this florid mendacity, this filth and criminality, this portraiture, and all the other trappings which aid the professional fraud to catch the dollars of the trusting public?

There are still other reasons why the physician may not advertise as the term is understood by the laity. Suppose he should say to the public that he has spent so many years in a certain university, where he received the foundation for a medical education, that he afterward took his medical degree in its department of medicine and surgery, then, after practicing general medicine ten or twelve years, he went to some large city, where he was a student of Professor Jones, the world's greatest specialist in a certain class of diseases, and that he was a resident here to practice Professor Jones' specialty? Now, all this would be unobjectionable if it did not appear as an advertisement in the foul company of the secular newspaper medical advertisers; but what good would it do the advertiser? The public never heard of Professor Jones, his name nor portrait never appeared in the papers, therefore his student must be a poor stick. Beside, have they not their own great specialists at their very doors, with a column of testimonials, headed by the rascal's portrait? The attitude of the public toward the medical profession and all things relating to cures is an anomaly.

They seem to have kept pace with all forms of learning except in medicine. Here the grotesque painted and befeathered medicine man is still with us. Here only are miracles worked, and the newspapers give us so many each week that the spirits of the patriarchs of old must blush with envy (if spirits ever blush). In all other departments of human activity the miracle is conspicuous by its absence. No thirsting Moses has in modern times struck the pinnacled rocks of the Bad Lands and caused a stream of pure water to flow therefrom, nor has any modern Jonah taken a summer vacation trip to Europe in the stomach of an accommodating whale, nor does anyone now turn water into wine, though the knight of the white apron has been suspected of turning wine into half water.

How long before the public will learn that the laws of health and disease are as inexorable as those which regulate the seasons and day and night. If your friend and brother, O public, is all but destroyed by the tubercle bacillus, and your family physician, who has been like a father to you, tells you that the grim reaper is near at hand, will you take him to the advertising miracle-working quack who has "never lost a case of consumption," and have his days made uncomfortable by the foolish bombastic rant of this fellow who wants only your money, and then have him insult you by telling you that your friend and brother was "not brought to me soon enough."

Why does not the lawyer advertise? He might say for instance, that he never lost a case in which he had defended a murderer, although he has had hundreds of such cases. How long would a judge permit such a lawyer to practice in his court? Would the public give heed to a lawyer of this kind, except to spit upon and spurn him? Yet the statement is not as absurd as that of the medical quack who advertises to the same purpose, and yet the learned judge is willing to give a signed testimonial, mayhap with his portrait, which Dr. Fraud proudly displays in his next advertisement.

What, O learned and incorruptible judge! has the noisy rascal whose nostrums you thus commend ever done for the cause of humanity? Do you know that he has invariably stolen a common instrument of the medical profession, dressed it in the garb of ancient mysticism, put the mask of miracle upon its face and sent it forth to rob under the pretense of doing good? Be lenient with the man who comes before you charged with getting money under false pretense, for you are accessory to the crime.

Why do not ministers of the gospel advertise? Using the language of the fraudulent medical advertiser, they might tell of new and improved ways of saving the soul, discovered by some pious man in Central Africa or the Fiji Islands. These reverend gentlemen might say that thousands of happy souls are in paradise to-day, which were all saved by their methods, and that they can give treatment by mail at $3 a week. I have before me an advertisement which contains the following, signed by a gentleman who writes "Reverend" as a part of his name. He declares that: "When I entered Dr. Fraud's Institute I had catarrh of the head, throat, stomach and bowels. Under Dr. Fraud's incomparable treatment I got entirely well. I was not only cured of my infirmities, including deafness, but completely re-established my health." Suppose now that the Rev. Jones used the same methods to get "trade" as Dr. Fraud uses, and suppose (which requires some imagination) that the Rev. Jones had converted by a "new method" Dr. Fraud, and made him an honest Christian gentlemen. It would be only proper and reciprocal for Dr. Fraud to give the following testimonials: "When I met the Rev. Jones I was a very bad man, I suffered with dry rot of the soul, lack of conscience, and the difference between truth and falsehood was not known to me. Indeed, so bad was my case that I had no remembrance of ever having known truth or honor. After remaining in the Rev. Jones' Hallelujah Institute and taking his salvation incantations and regenerating rubbings, I became again a good man and promise to give back as far as possible all that I have got by fraud. I shall advise all persons similarly afflicted to go to Rev. Jones' Institute." If the Rev. Jones is shocked because things sacred to him are thus treated flippantly, let him be told that there are no things in our lives more sacred than truth, honor and charity. How much of any of these virtues is possessed by the fraud whom he is helping to continue in this shameless imposition upon the people.

When will our lawmakers compel those debased men and women who so lightly trifle with valuable human lives, to give satisfactory proof of the truth of their claims, or to be at once suppressed? Honorable members of the medical profession are every day doing deeds, which, when compared to the silly gabble of the blatant fraud, shine as the electric arc light to the penny rush. They are forming ties which bind them to beautiful, intelligent and loving households, who give to them the priceless honor of gratitude and confidence which no other man receives, and in no other profession or calling does a member receive the ungrudging honor of his fellows for meritorious deeds more than in the medical profession.

